# Emerging roles of CAR-NK cell therapies in tumor immunotherapy: current status and future directions

**DOI:** 10.1038/s41420-024-02077-1

**Published:** 2024-07-10

**Authors:** Yan Zhong, Jingfeng Liu

**Affiliations:** 1 Department of Pathology, Shenzhen Hospital, Southern Medical University, Shenzhen, China; 2 Shenzhen Key Laboratory of Immunity and Inflammatory Diseases, Peking University Shenzhen Hospital, Shenzhen, China; 3 Institute of Biomedicine and Biotechnology, Shenzhen Institute of Advanced Technology, Chinese Academy of Science, Shenzhen, China

**Keywords:** Cancer immunotherapy, Translational research

## Abstract

Cancer immunotherapy harnesses the body’s immune system to combat malignancies, building upon an understanding of tumor immunosurveillance and immune evasion mechanisms. This therapeutic approach reactivates anti-tumor immune responses and can be categorized into active, passive, and combined immunization strategies. Active immunotherapy engages the immune system to recognize and attack tumor cells by leveraging host immunity with cytokine supplementation or vaccination. Conversely, passive immunotherapy employs exogenous agents, such as monoclonal antibodies (anti-CTLA4, anti-PD1, anti-PD-L1) or adoptive cell transfers (ACT) with genetically engineered chimeric antigen receptor (CAR) T or NK cells, to exert anti-tumor effects. Over the past decades, CAR-T cell therapies have gained significant traction in oncological treatment, offering hope through their targeted approach. However, the potential adverse effects associated with CAR-T cells, including cytokine release syndrome (CRS), off-tumor toxicity, and neurotoxicity, warrant careful consideration. Recently, CAR-NK cell therapy has emerged as a promising alternative in the landscape of tumor immunotherapy, distinguished by its innate advantages over CAR-T cell modalities. In this review, we will synthesize the latest research and clinical advancements in CAR-NK cell therapies. We will elucidate the therapeutic benefits of employing CAR-NK cells in oncology and critically examine the developmental bottlenecks impeding their broader application. Our discussion aims to provide a comprehensive overview of the current status and future potential of CAR-NK cells in cancer immunotherapy.

## Facts

- CAR-NK therapy emerges as a promising tumor immunotherapy alternative with enhanced safety and therapeutic effects. This review comprehensively makes a comparison between CAR-T and CAR-NK cell therapies to provide deep understanding of CAR-NK cell biological characteristics.
- This review highlights the recent progress of CAR-NK research and their clinical applications, focusing on the future directions in breaking tumor immunosuppressive microenvironments in solid tumors.

## Open questions

- Despite the promise of CAR-NK cell therapy, several open questions remain. A primary concern is the persistence and long-term efficacy of CAR-NK cells, as they do not proliferate as extensively as CAR-T cells, potentially limiting their duration of tumor control. The identification of optimal target antigens to maximize efficacy while minimizing off-target effects is another area of active research. Additionally, the scalability and consistency of manufacturing clinically effective CAR-NK cells are challenges that need to be addressed. Furthermore, the integration of CAR-NK therapy with other treatments, such as checkpoint inhibitors or conventional chemotherapy, to improve outcomes is an area ripe for exploration.

## Introduction

Cellular immunotherapy has revolutionized the approach to cancer treatment, marking a departure from conventional therapies by harnessing the body’s own immune system to combat malignancies. Central to this revolution is the development of chimeric antigen receptor (CAR) technology, which enables the genetic modification of immune cells to specifically target and destroy cancer cells [1]. CAR constructs are synthetic molecules designed to combine an extracellular antigen recognition domain, typically derived from a monoclonal antibody, with intracellular signaling domains that activate the immune cells upon antigen engagement [2–4]. This design allows T cells, when engineered to express CARs (CAR-T cells), to recognize and attach to specific antigens present on the surface of tumor cells, leading to their selective destruction [5–8].

The clinical application of CAR-T cell therapy has yielded remarkable success in treating certain hematological malignancies, such as relapsed or refractory B-cell acute lymphoblastic leukemia (ALL), diffuse large B-cell lymphoma (DLBCL) and multiple myeloma [9, 10]. FDA-approved therapies like tisagenlecleucel (tisa-cel) and axicabtagene ciloleucel (axi-cel) have demonstrated significant efficacy in these settings, offering hope to patients who have exhausted other treatment options [11–13]. However, the clinical journey of CAR-T cell therapy has also uncovered challenges, including the management of severe side effects such as cytokine release syndrome (CRS) and neurotoxicity, as well as the complexity of manufacturing and delivering these personalized therapies.

**Fig. 1:**
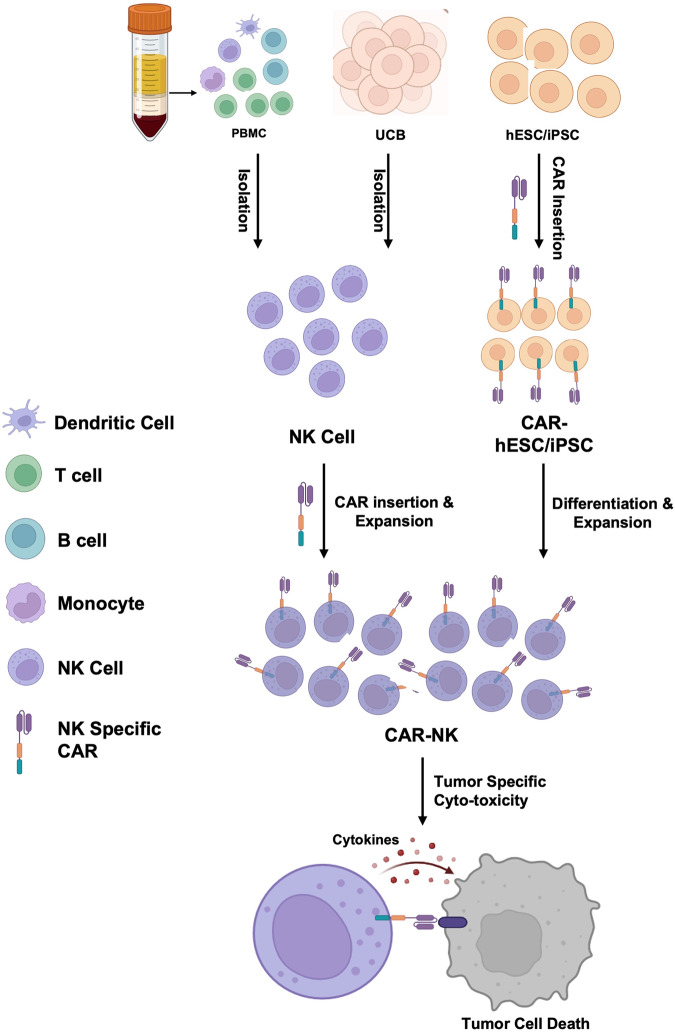
Schematic overview of the generation and application of CAR-NK therapy. Primary NK cells are isolated from a suitable source, which could be autologous or allogeneic donors’ peripheral blood and umbilical cord blood. The selected NK cells are genetically modified to express chimeric antigen receptors (CARs) through using viral vectors (such as retroviruses or lentiviruses) for stable gene delivery. Or, CAR-NK cells can be differentiated from CAR modified human embryonic stem cell (hESC) or induced pluripotent stem cell (iPSC). The genetically modified NK cells undergo in vitro expansion and activation. The prepared CAR-NK cells are infused into the patient and mediate direct antitumor effects through target cell recognition, leading to the release of cytolytic granules that induce death in the tumor cells.

Building upon the success and learning from the limitations of CAR-T cell therapy, researchers have turned their attention to natural killer (NK) cells. As part of the innate immune system, NK cells possess intrinsic anti-tumor properties without the need for prior sensitization, making them promising candidates for CAR engineering (CAR-NK cells) [14–16]. By combining the specificity of CAR targeting with the natural cytotoxicity of NK cells, CAR-NK therapies aim to offer a safer and potentially more universally applicable form of cellular immunotherapy (Fig. 1).

As integral members of the innate immune system, NK cells are characterized by their rapid response to malignant transformations and viral infections [17]. Their cytotoxic action is executed through the deployment of perforin and granzymes, leading to the apoptosis of targeted cells. This activity is modulated by a complex array of activating and inhibitory receptors on their surface, which recognize alterations in the expression of major histocompatibility complex (MHC) class I molecules on potential target cells. NK cells also possess a diverse set of activating receptors, such as NKG2D and the natural cytotoxicity receptors (NCRs), which detect stress-induced ligands. This dynamic interplay enables NK cells to discern and eliminate abnormal cells while sparing healthy ones. Additionally, NK cells are prolific cytokine producers, secreting substances like IFN-γ and TNF-α that modulate the immune response, enhance antigen presentation, and directly inhibit tumor cell proliferation or induce apoptosis.

Despite their potential, the clinical application of CAR-NK cell therapy, especially in solid tumors, is met with complex challenges [18–21]. The immunosuppressive tumor microenvironment (TME) poses a formidable barrier, often neutralizing the efficacy of immunotherapeutic agents. In this review, we aim to provide a comprehensive overview of the current status of CAR-NK cell therapies, emphasizing their biological underpinnings and advantages over other forms of immunotherapy. We will explore state-of-the-art techniques in CAR-NK cell generation and discuss clinical progress and setbacks. A focal point will be identifying and addressing the bottlenecks that impede the full potential of CAR-NK therapies in solid tumors, setting the stage for future advancements in this promising field.

## Current status of CAR-NK therapy

The therapeutic landscape of cancer has been substantially reshaped by the advent of CAR-NK cell therapies. Recent clinical trials have begun to unveil the clinical effects and potential of these novel treatments, marking a new chapter in the field of immunotherapy. In recent years, a growing number of studies have evaluated CAR-NK cells in various malignancies [22, 23]. Notably, a pivotal phase I/II trial (NCT03056339) conducted at the MD Anderson Cancer Center demonstrated that CAR-NK cells derived from cord blood could be safely administered to patients with relapsed or refractory CD19-positive cancers, including non-Hodgkin’s lymphoma and chronic lymphocytic leukemia [24]. The trial reported a promising response rate with minimal adverse events, highlighting the potential for broader application. Another significant trial (NCT03383978) investigating the use of CAR-NK cells targeting the HER2 antigen in neuroblastoma showcased not only safety but also signs of efficacy in a solid tumor setting, an area where CAR-T cells have faced challenges [25]. By searching “CAR-NK cells” in “ClinicalTrials.gov”, we can hit 50 results in last 5 years and these ongoing trials have fueled optimism for CAR-NK therapy’s applicability across a spectrum of cancers, including solid tumors (Table 1). The current status of CAR-NK therapy is one of cautious optimism. With several clinical trials underway and many more in the pipeline, the field is rapidly expanding our understanding of how these therapies can be harnessed to combat cancer effectively. The clinical effects observed thus far provide a compelling argument for the continued investigation and development of CAR-NK cells as a cornerstone of future immunotherapeutic strategies.

**Table 1:** Ongoing clinical trials targeting solid tumors using CAR-NK.

NCT Number	Cell Source	Tumor Type	Tumor Antigen	Phase Stage
NCT05776355	N/A	Ovarian Cancer	NKG2D	N/A
NCT05213195	N/A	Refractory Metastatic Colorectal Cancer	NKG2D	Phase 1
NCT05410717	PBMC	Stage IV Ovarian Cancer, Refractory Testis Cancer, Endometrial Cancer Recurrent	CLDN6, GPC3, Mesothelin, AXL	Phase 1
NCT06066424	Cord Blood	Non-small Cell Lung Cancer, Breast Cancer	TROP2	Phase 1
NCT05507593	N/A	Small Cell Lung Cancer	DLL3	Phase 1
NCT04847466	NK-92	Gastric or Head and Neck Cancer	PD-L1	Phase 2
NCT05922930	Cord Blood	Ovarian Cancer, Adenocarcinoma, Pancreatic Cancer	TROP2	Phase 1/2
NCT05703854	Cord Blood	Advanced Renal Cell Carcinoma, Mesothelioma Osteosarcoma	CD70	Phase 1/2

## Superiority of CAR-NK cell therapy

As the field of cellular immunotherapy matures, the quest for safer and more practical treatments has brought CAR-NK cells into the spotlight. Their emergence is shadowed by the success of CAR-T cells, which, despite their efficacy, are often associated with significant toxicities that can limit their use and challenge patient management. In this context, we explored a thorough comparison between CAR-T and CAR-NK therapy (Table 2). Additionally, we discuss the superiority of CAR-NK cells in two key areas: reduced side effects and the advantage of their “off-the-shelf” nature.

**Table 2:** Comparison between the pros and cons of CAR-T and CAR-NK cell therapies.

Aspect	CAR-T Therapy	CAR-NK Therapy
Cell Source	Autologous: Patient-derived, risk of variable quality. Allogeneic: Donor-derived, risk of GvHD.	Allogeneic: Derived from donors or NK cell lines like NK-92, less risk of GvHD, more consistent product.
Efficacy	High efficacy in B-cell malignancies (e.g., CD19-targeted CAR-T for ALL).	Emerging data show efficacy in hematological malignancies; solid tumors are an active area of research.
Persistence	Long-lived with potential for durable responses. Clonal expansion can lead to long-term remissions.	Shorter lifespan; research into cytokine support (e.g., IL-15) to enhance persistence is ongoing.
CRS	Severe CRS due to massive T cell activation; requires careful management.	Lower incidence and severity of CRS due to different activation and signaling pathways.
GvHD	Significant concern with allogeneic CAR-T cells, necessitating immune matching or gene editing.	Reduced risk due to lack of T cell receptor (TCR) and lower likelihood of causing GvHD.
Manufacturing	Time-consuming, complex, personalized manufacturing; limited scalability.	Potential for ‘off-the-shelf’ use, easier manufacturing, better scalability.
Heterogeneity	T cells can undergo extensive differentiation, affecting potency and function.	NK cells are less prone to differentiation-induced functional changes, leading to a more uniform product.
TME Resistance	Susceptible to immunosuppressive TME; may require additional modification (e.g., PD-1 knockout).	Innate resistance to TME suppression; research into enhancing TME infiltration is ongoing (e.g., chemokine receptor modification).
On-Target, Off-Tumor Toxicity	Can be severe due to target antigen presence on healthy cells (e.g., CD19 on normal B cells).	Typically less severe; NK cells have innate killing mechanisms that may reduce risk.
Preconditioning	Often requires lymphodepleting chemotherapy with its own risks and side effects.	May require less intensive preconditioning, lowering overall treatment toxicity.
CAR Construct	Composed of an antigen-recognition domain, hinge region, transmembrane domain, and intracellular signaling domains (e.g., CD3ζ along with CD28 and/or 4–1BB co-stimulatory domains). Known to induce strong T cell activation and proliferation.	Similar structure but may include NK cell-specific signaling domains (e.g., DAP10 or DAP12) to optimize NK cell activation and function. Research into adding NKG2D, DNAM-1 or 2B4 co-stimulatory domains is underway to enhance their efficacy.

### Reduced side effects compared to CAR-T cells

One of the main concerns with CAR-T cell therapy is the occurrence of severe side effects, such as cytokine release syndrome (CRS) and neurotoxicity. CRS is a systemic inflammatory response caused by the release of large amounts of cytokines by activated T cells. Upon CAR-T cell engagement with target cancer cells, an intense activation occurs, leading to the rapid proliferation of these engineered cells and the subsequent release of pro-inflammatory cytokines. This cytokine storm, featuring elevated levels of IL-6, IFN-γ, TNF-α, and others, triggers a cascade of immune responses that recruit additional immune cells into the fray. The excessive cytokine levels then precipitate a systemic inflammatory response, manifesting in symptoms ranging from mild fever to severe multi-organ dysfunction. CRS occurs in a significant proportion of patients treated with CAR-T cell therapies, with severe CRS occurring in up to 30% of cases, depending on the specific CAR-T construct and the disease being treated [26–29]. Treatment for alleviating CRS includes the administration of tocilizumab (an IL-6 receptor antagonist) and corticosteroids, which can be effective but may also dampen the antitumor effects of the CAR-T cells [30]. From the early clinical data, they suggest that CRS is less common and less severe with CAR-NK cell therapies [31]. Unlike T cells, the inherent immunological properties of NK cells provide a higher activation threshold and do not require antigen-specific activation, leading to a more controlled cytokine release upon encountering target cells. NK cells inherently lack the robust proliferative response seen in T cells, curtailing the magnitude of cytokine secretion. Furthermore, their cytotoxic action is predominantly direct and does not depend extensively on cytokine-mediated pathways but involve a complex interplay of activating and inhibitory receptors due to the balance of NCRs (NKp30, NKp44, NKp46, etc.) and KIRs. The milder nature of CRS with CAR-NK cells may reduce the need for intensive management strategies, potentially allowing for outpatient treatment.

Neurotoxicity, another serious complication during CAR-T treatment, presents with symptoms ranging from confusion to severe seizures and cerebral edema, which has been observed in up to 50% of patients receiving certain CAR-T cell therapies [26, 27]. Elevated cytokines following CAT-T infusion compromise the blood-brain barrier (BBB), enabling immune cells, including activated CAR-T cells, to infiltrate the central neuro system (CNS). This infiltration can directly provoke neuroinflammation. Concurrently, these cytokines activate microglia, the brain’s resident immune cells, exacerbating neuroinflammatory responses. The resulting endothelial cell activation within the CNS further disrupts the BBB’s integrity, which generates a positive feedback loop to further worsening neuronal damage. In contrast to CAR-T cells, CAR-NK cells have not been associated with significant neurotoxicity in early clinical trials due to their innate characteristics with a reduced capacity for cytokine production and limited proliferation in vivo which curtails extensive immune cell infiltration into the CNS [32]. Collectively, CAR-NK cells have demonstrated a significantly lower risk of such side effects. The innate biology of NK cells contributes to this safety profile.

### “Off-the-shelf” potential

The safety advantages of CAR-NK cells are complemented by their potential as an “off-the-shelf” therapy [33–37]. Unlike CAR-T cells, which require patient-specific manufacturing that is both time-consuming and costly, CAR-NK cells can be derived from various sources, such as umbilical cord blood or induced pluripotent stem cells (iPSCs), allowing for the creation of a ready-to-use therapeutic product. This approach could significantly reduce the time between diagnosis and treatment, a crucial factor for patients with aggressive malignancies. Additionally, precision genome editing could be applied during iPSC stage to obtain gain-of-function attributes in the resulting CAR-NK cell products. There are plenty of reports discussed the feasibility of such technology. For instance, Dan Kaufman group established human CISH-knockout NK cell platform via CISH-knockout iPSC which demonstrated increased IL-15 signaling activity [38]. This modification can potentially create an “armored” CAR-NK cell that has improved persistence and efficacy in vivo. Moreover, the “off-the-shelf” nature of CAR-NK therapy aligns with the move towards scalable production processes in cellular therapies. It opens the possibility for broader access to treatment, reduced costs, and improved consistency in product quality. The ability to pre-manufacture and store CAR-NK cells also enables immediate retreatment, if necessary, an option not readily available with patient-specific CAR-T therapies.

In conclusion, CAR-NK cell therapy holds a distinct advantage over CAR-T cell therapy in terms of a more favorable safety profile and the practicality of an “off-the-shelf” approach. These attributes not only enhance the appeal of CAR-NK cells but also address some of the critical challenges faced by current CAR-T cell therapies. As clinical experiences with CAR-NK cells expand, their potential to become a mainstay in cancer immunotherapy becomes increasingly evident.

## Generation of CAR-NK cell

The successful generation of CAR-NK cells is pivotal to their therapeutic efficacy and is influenced by the CAR construct design, the source of NK cells, and the manufacturing technology. Recent advancements have led to the development of CAR constructs that are specifically tailored to NK cell biology, enhancing their activation and cytotoxicity against cancer cells.

### NK cell specific CAR structure

The structure of a CAR-NK cell is designed to capitalize on the unique activation mechanisms of NK cells. According to research led by Dan Kaufman and colleagues, the CAR construct typically comprises an extracellular antigen recognition domain, a transmembrane domain, and an intracellular signaling domain [39]. Unlike CAR-T cells, the intracellular domain in CAR-NK cells often includes signaling motifs from NK cell activating receptors, such as DAP12 or CD3ζ, coupled with co-stimulatory domains from 2B4 (CD244) or DNAM-1 (CD226) to enhance activation and cytotoxic function [3, 18]. This configuration is crucial as it leverages the natural cytotoxic pathways of NK cells, ensuring that the engineered cells function optimally within their innate immune response mechanisms.

Recently, mounting progress has been reached for CAR structure design. For instance, Bi-specific CAR-NK cells are engineered to recognize two different tumor antigens simultaneously. This approach aims to improve tumor targeting and minimize the risk of tumor escape due to antigen loss or heterogeneity. For example, a bi-specific CAR could target both CD19 and CD22 on B-cell malignancies, increasing the likelihood of CAR-NK cell engagement with cancer cells [40]. Moreover, Logic-gated CARs are designed to require multiple signals for activation, which can potentially reduce off-tumor toxicity by ensuring that CAR-NK cells are only fully activated in the presence of a specific combination of antigens found on tumor cells. For instance, an AND-gate CAR requires the presence of two antigens to trigger NK cell activation, while an OR-gate CAR can be activated by either one of two antigens [41]. Similarly, SynNotch receptors are a type of synthetic receptor that can be used to control the specificity and activation of CAR-NK cells [42]. These receptors can be designed to respond to one antigen by expressing a CAR for a second antigen, thereby creating a two-step recognition process that enhances specificity for tumor cells. Collectively, these advanced CAR designs are part of ongoing research efforts to create more effective and safer CAR-NK cell therapies for cancer treatment. As this field is rapidly advancing, continuous monitoring of the latest scientific literature is essential for up-to-date information on novel CAR structures and their clinical applications.

### Sources of CAR-NK cells

The selection of a suitable source for CAR-NK cells is a critical step in the development of effective therapies. CAR-NK cells can be derived from various sources, each with its own set of advantages.

#### PBMC derived CAR-NK

Peripheral blood mononuclear cells (PBMCs) derived NK cell is a convenient source due to its easy accessibility. Autologous NK cells can be harvested from a patient’s own blood, minimizing the risk of immune rejection, while allogeneic NK cells can be collected from healthy donors and have the potential for use in multiple patients. There are well-established clinical protocols for the isolation and expansion of NK cells from peripheral blood, which can be leveraged for the production of CAR-NK cells. However, the quantity and functionality of NK cells can vary greatly between individuals and may be affected by factors such as age, health status, and prior treatments, especially in cancer patients. Additionally, the relatively low frequency of NK cells in peripheral blood necessitates the use of cell expansion technologies to generate sufficient numbers for therapeutic purposes. Although the advancements in the use of feeder cell lines that express membrane-bound IL-21 (miL-21) has been shown to enhance the expansion and function of NK cells [43], ex vivo expansion can lead to NK cell exhaustion, which may reduce their effectiveness. Researchers are further exploring various cytokine cocktails and feeder cells to optimize the expansion and activation of NK cells to maintain their cytotoxic function. For instance, researchers have been investigating the timed addition of various cytokines during the expansion process to improve the persistence and antitumor activity of CAR-NK cells derived from PBMC [44, 45]. In summary, while peripheral blood is a practical and widely used source for generating CAR-NK cells, there is ongoing research aimed at overcoming its limitations to enhance the yield, potency, and durability of these immune effector cells for therapeutic applications.

#### UCB derived CAR-NK

Umbilical cord blood (UCB) represents another significant source for the derivation of NK cells used in the generation of CAR-NK cell therapies due to several key attributes. One of the primary advantages of UCB-derived NK cells is their relative immaturity compared to adult peripheral blood NK cells. This immaturity translates into a higher proliferative capacity upon activation, making them a potent candidate for cellular therapies. Furthermore, these immature NK cells tend to exhibit a reduced risk of causing GvHD, which is highly beneficial in the context of allogeneic transplantation. The diverse ethnic representation within UCB banks enhances the potential for finding suitable matches for patients from various backgrounds, which is crucial for the broad applicability of CAR-NK cell therapies. Additionally, UCB-derived NK cells have shown a high potential for ex vivo expansion, enabling the generation of large numbers of cells necessary for therapeutic dosing. However, this expansion process can be time-consuming and may delay treatment for patients in urgent need. Researchers are actively working to overcome this limitation by developing and refining protocols for the ex vivo expansion and activation of UCB-derived NK cells. These protocols often involve the use of cytokines such as IL-15 or a combination of IL-15, IL-21 and stem cell factor (SCF) to promote cell growth while maintaining cytotoxic capabilities [46]. Advances in gene editing technologies have also enabled more efficient insertion of CAR constructs into UCB-derived NK cells, enhancing their specificity and killing capacity against target cancer cells [47–51]. Despite the challenges, the ongoing optimization of techniques for the cultivation and genetic modification of UCB-derived NK cells continues to solidify their role as a valuable resource in the development of innovative immunotherapies.

#### hESC or iPSC derived CAR-NK

Human embryonic stem cells (hESCs) and induced pluripotent stem cells (iPSCs) offer exciting avenues for the derivation of CAR-NK cells, each with unique advantages and challenges [16, 32, 52–55]. Both hESCs and iPSCs have the inherent ability to differentiate into any cell type, including NK cells, providing a potentially unlimited source of cells for therapeutic applications. iPSCs, in particular, can be derived from adult somatic cells, allowing for the creation of patient-specific or universally compatible cell lines depending on the genetic modifications applied. The use of hESCs and iPSCs for CAR-NK cell therapy is advantageous due to the consistent and renewable supply of cells that can be generated. This is particularly beneficial for scaling up production to meet clinical demand. Additionally, the ability to engineer these cells at the pluripotent stage allows for precise genetic modifications, such as the insertion of CAR constructs or the knockout of genes that encode inhibitory receptors, which can enhance the efficacy and safety of the resulting NK cells [38]. However, the use of hESCs is fraught with ethical concerns due to their derivation from early human embryos. These ethical considerations limit their use in some countries and can complicate the regulatory approval process. iPSCs, while circumventing these ethical issues, still present significant technical challenges [56]. The reprogramming process to generate iPSCs can be inefficient and time-consuming, and there is a risk of introducing genetic abnormalities during this process that could potentially lead to tumorigenesis [57]. Moreover, the differentiation of hESCs and iPSCs into functional NK cells is a complex and tightly regulated process that requires a deep understanding of developmental cues. Researchers must replicate these cues in vitro to guide the stem cells through a multi-stage differentiation process that yields fully functional NK cells. This process is not only technically demanding but also requires stringent quality control measures to ensure the safety and consistency of the therapeutic product. Despite these challenges, recent advancements in stem cell technology have led to more efficient and reliable methods for differentiating hESCs and iPSCs into NK cells. For example, specific culture conditions that mimic the natural developmental environment of NK cells have been optimized, and the use of small molecules to NK cell maturation has shown promise in improving anti-cancer efficiency [58–60]. With continued research and development, hESC- and iPSC-derived CAR-NK cells have the potential to become a mainstay in the field of cellular immunotherapy, offering treatments that are both effective against cancer and customizable to individual patient needs.

## Bottlenecks of CAR-NK cell in solid tumor

The transition of CAR-NK cell therapy from hematological malignancies to solid tumors has been met with considerable challenges. Despite their promise, several biological and technical hurdles have emerged, complicating the effective deployment of CAR-NK cells against solid cancers.

### Tumor immunosuppressive microenvironment

The tumor microenvironment (TME) is characterized by a highly immunosuppressive milieu that can significantly hinder the efficacy of CAR-NK cell therapies. Within the TME, a variety of cells, including regulatory T cells (Tregs), myeloid-derived suppressor cells (MDSCs), cancer-associated fibroblasts (CAFs), and a host of immunosuppressive cytokines like TGF-β, create a barrier to immune cell infiltration and function [37, 61–64]. Recent research has shown that these elements can modulate NK cell receptors and signaling pathways, dampening the cytotoxic response of CAR-NK cells. For example, TGF-β has been found to downregulate the expression of NKG2D, an activating receptor on NK cells, thus reducing their ability to recognize and kill tumor cells [65]. iL-10 secreted by Treg and MDSCs can inhibit the function of NK cells and other effector cells, thus reducing their cytotoxic activity against tumor cells [62]. Furthermore, indoleamine 2,3-dioxygenase (IDO1) is an enzyme expressed by some tumors that catabolizes tryptophan into kynurenine, leading to the suppression of NK cell function and the promotion of Treg cell development, further dampening the immune response [66, 67].

To counteract this immunosuppression, strategies such as co-administering checkpoint inhibitors or blocking TGF-β signaling are being explored. For instance, engineering CAR-NK cells to secrete cytokines like IL-15 can enhance their survival and promote an inflammatory TME that is more conducive to NK cell activity [68]. Another approach is to modify CAR-NK cells to express a dominant-negative TGF-β receptor or TGF-β “trap” molecules, thereby preventing the inhibitory signaling induced by the TME [69]. Additionally, the use of pharmacological inhibitors of IDO1, such as epacadostat, has been proposed to prevent the depletion of tryptophan in the TME and enhance NK cell function [70, 71]. Recent research has also focused on genetically modifying CAR-NK cells to knock out NKG2A or to incorporate a checkpoint blockade molecule into the CAR construct itself. This would render the CAR-NK cells resistant to the inhibitory signals within the TME. An example of this is a study by Liu et al., which showed that NKG2A knockout CAR-NK cells displayed enhanced anti-tumor activity in a preclinical model [24, 72].

### Persistence of CAR-NK cells

The physiological basis for the limited persistence of CAR-NK cells in the context of solid tumors is multifaceted. Typically, NK cells have a short lifespan and a rapid turnover rate in vivo, which are natural characteristics that distinguish them from longer-lived lymphocytes like T cells [17]. In the tumor setting, the harsh conditions of the microenvironment, including hypoxia, nutrient deprivation, and exposure to immunosuppressive factors such as prostaglandin E2 (PGE2) and adenosine, further exacerbate the challenge by promoting NK cell apoptosis and dysfunction [63]. Moreover, the activation and cytotoxicity of NK cells are tightly regulated by a balance of activating and inhibitory signals. Persistent activation can lead to a state of exhaustion, similar to that observed in T cells, characterized by decreased functionality and survival.

Recent research progress has been aimed at enhancing the persistence of CAR-NK cells through various strategies. One approach is the genetic modification of CAR-NK cells to express cytokines that support their growth and survival. For instance, IL-15 is known to play a critical role in the development, survival, and activation of NK cells. Studies have shown that CAR-NK cells engineered to express IL-15 have improved persistence and antitumor activity in vivo [68]. Another strategy is to target apoptotic signals within tumor cells or to use gene-editing tools to knock out pro-apoptotic genes in CAR-NK cells [73]. This could potentially increase their resistance to the apoptotic signals within the TME. Additionally, modifying the CAR construct to include co-stimulatory domains that can provide potent survival signals upon engagement with their target antigen has been shown to enhance CAR-NK cell persistence.

To address NK cell exhaustion, researchers are investigating checkpoint blockade therapies that target inhibitory receptors on NK cells. For example, inhibiting TIGIT with monoclonal antibodies has been proposed to rejuvenate exhausted NK cells and prolong their functional activity within tumors. In terms of clinical research, a study by Zhu et al. demonstrated that a memory-like phenotype in CAR-NK cells could be induced through pre-activation with certain cytokines, leading to enhanced persistence and antitumor efficacy in vivo [38]. These memory-like CAR-NK cells exhibited a gene expression profile associated with longer life span and robust functionality compared to conventional NK cells.

### Tumor antigen escape

Tumor antigen escape is a significant hurdle in the success of CAR-NK cell therapy. Tumors can downregulate or lose expression of the target antigen, rendering CAR-NK cells ineffective. This phenomenon is underpinned by the genetic instability and heterogeneity often seen in solid tumors. To address this challenge, recent research has focused on developing CAR-NK cells with dual or even multiple antigens targeting capabilities. By recognizing more than one antigen, these multi-specific CAR-NK cells can reduce the likelihood of tumor escape due to antigen loss.

Physiologically, the selective pressure exerted by CAR-NK cells can lead to an outgrowth of antigen-negative tumor variants. To counter this, bi-specific or tri-specific CAR constructs have been developed. For instance, a bispecific CAR that targets both HER2 and MUC1 can engage different populations within a heterogeneous tumor mass, providing broader coverage and reducing the risk of escape variants dominating.

In summary, while CAR-NK cells hold significant potential for the treatment of solid tumors, addressing these bottlenecks requires a multifaceted approach that combines advanced genetic engineering, innovative combination therapies, and a deeper understanding of tumor biology. Overcoming these obstacles will be crucial for realizing the full potential of CAR-NK cell therapy in solid tumors.

## Conclusion remarks

Throughout this review, we have explored the innovative realm of CAR-NK cell therapy, a burgeoning frontier in the fight against cancer. The unique attributes of NK cells, equipped with CARs, hold significant promise for the treatment of both hematological malignancies and solid tumors. We have dissected the structure and generation of CAR-NK cells, highlighted their sources, and embraced the transformative potential of iPSC-derived CAR-NK cells. However, the path to realizing the full potential of CAR-NK therapy in solid tumors is fraught with challenges. We have identified critical bottlenecks, such as the immunosuppressive tumor microenvironment, the limited in vivo lifespan of CAR-NK cells, and the specificity of tumor antigens. These hurdles underscore the complexity of the tumor battleground and the resilience of malignancies to immune-mediated interventions.

Looking forward, we remain optimistic to navigate these obstacles. Strategies to enhance the viability and persistence of CAR-NK cells post-transplantation, such as cytokine support and genetic armoring, are under active investigation. The synergistic use of immune checkpoint inhibitors with CAR-NK therapy is another exciting avenue that could potentiate anti-tumor responses. Additionally, targeting the tumor microenvironment directly and improving antigen specificity are promising strategies that could refine the precision and efficacy of CAR-NK cell therapy.

In conclusion, while challenges persist, the advancements in CAR-NK cell therapy represent a paradigm shift in cancer treatment. The continuous evolution of this modality, fueled by cutting-edge research and clinical innovation, holds great hope for delivering new therapeutic options to patients battling cancer.
